# Delivering transcutaneous auricular neurostimulation (tAN) to improve symptoms associated with opioid withdrawal: results from a prospective clinical trial

**DOI:** 10.1186/s42234-022-00095-x

**Published:** 2022-08-18

**Authors:** Carlos F. Tirado, Stephanie N. Washburn, Alejandro Covalin, Caroline Hedenberg, Heather Vanderpool, Caroline Benner, Daniel P. Powell, Melanie A. McWade, Navid Khodaparast

**Affiliations:** 1 CARMAhealth Management, Inc., 630 W 34th St #301, Austin, TX 78705 USA; 2 Spark Biomedical, Inc., 18208 Preston Road, Ste D9-531, Dallas, TX 75252 USA

**Keywords:** Transcutaneous auricular neurostimulation, Vagus nerve stimulation, Trigeminal nerve stimulation, Opioid withdrawal symptoms, Non-opioid treatment, Addiction

## Abstract

**Background:**

As pharmacological treatments are the primary option for opioid use disorder, neuromodulation has recently demonstrated efficacy in managing opioid withdrawal syndrome (OWS). This study investigated the safety and effectiveness of transcutaneous auricular neurostimulation (tAN) for managing OWS.

**Methods:**

This prospective inpatient trial included a 30-minute randomized, sham-controlled, double-blind period followed by a 5-day open-label period. Adults with physical dependence on opioids were randomized to receive active or sham tAN following abrupt opioid discontinuation. The Clinical Opiate Withdrawal Scale (COWS) was used to determine withdrawal level, and participants were required to have a baseline COWS score ≥ 13 before enrollment. The double-blind period of the study occurred during the first 30-minutes to assess the acute effects of tAN therapy compared to a sham control. Group 1 received active tAN during both the 30-minute double-blind period and the 5-day open-label period. Group 2 received passive sham tAN (no stimulation) during the double-blind period, followed by active tAN during the 5-day open-label period. The primary outcome was change in COWS from baseline to 60-minutes of active tAN (pooled across groups, accounting for 30-minute delay). Secondary outcomes included difference in change in COWS scores between groups after 30-minutes of active or sham tAN, change in COWS scores after 120-minutes of active tAN, and change in COWS scores on Days 2–5. Non-opioid comfort medications were administered during the trial.

**Results:**

Across all thirty-one participants, the mean (SD) COWS scores relative to baseline were reduced by 7.0 (4.7) points after 60-minutes of active tAN across both groups (*p* < 0.0001; Cohen’s d = 2.0), demonstrating a significant and clinically meaningful reduction of 45.9%. After 30-minutes of active tAN (Group 1) or sham tAN (Group 2), the active tAN group demonstrated a significantly greater COWS score reduction than the sham tAN group (41.7% vs. 24.1%; *p* = 0.036). Participants across both groups achieved an average COWS reduction up to 74.7% on Days 2–5.

**Conclusion:**

Results demonstrate tAN is a safe and effective non-opioid approach for reducing symptoms of OWS. This study supported an FDA clearance.

**Clinical trial registration:**

clinicaltrials.gov/ct2/show/NCT04075214, Identifier: NCT04075214, Release Date: August 28, 2019.

## Background

The United States is experiencing an epidemic for prescription and non-prescription opioids, which have continued to rise since the 1990s. During 2015, approximately 2.1 million Americans were severely dependent to prescription opioids, and 513,000 to heroin (Kolodny et al. 2015). In 2020, the Center for Disease Control reported 93,331 substance use overdose deaths (Ahmad et al. 2021). The continuing increase in opioid-related deaths from 2015 (18%) to 2020 (60%) may partly be attributed to the mental health crisis during the Covid-19 pandemic (Baumgartner and Radley 2021). Aside from pain mitigation, opioids may provide motivation behind drug-seeking behavior of dependent individuals. This not only attributes to positive reinforcement derived from the euphoric effects, but also from negative reinforcement derived from the withdrawal symptoms that result from cessation. The emergence of opioid withdrawal syndrome (OWS) can be a significant barrier for dependent individuals to cease opioid consumption (Pantazis et al. 2021). As such, there is a need for a non-opioid intervention to mitigate symptoms of OWS.

Alternative approaches for treating OWS and opioid use disorder (OUD) are a major priority for government agencies given the substantial impact on health, social, and economic welfare. Pharmacotherapies are the primary treatment for OUD. Psychosocial and behavioral adaptation approaches may also be administered alone or in combination with pharmacotherapy. Commonly use pharmacotherapies for OUD include methadone, buprenorphine, and naltrexone (Stotts et al. 2009). Methadone and buprenorphine are semi-synthetic opioid derivatives that bind to opioid receptors, allowing individuals to discontinue the misuse of opioids (Ibrahim et al. 2000). Naltrexone is an opioid-antagonist, which blocks the opioid receptors, and prevents opioids from binding (Comer et al. 2006). In 2018 the FDA approved lofexidine hydrochloride (Lucemyra), the first non-opioid medication for OWS in adults.

Despite strong evidence supporting the use of buprenorphine and methadone (Johnson et al. 2000), the Substance Abuse and Mental Health Services Administration reported evidence from 2015 that of the 21.7 million Americans that needed treatment, only 2.3 million received pharmacotherapy for OUD. (Park-Lee et al. 2018). A primary constraint on access to pharmacotherapy for OUD is the limited availability of physicians and clinics able to provide controlled opioid-based pharmacotherapies (Amiri et al. 2021). In addition to regulatory reform to expand access to opioid-based pharmacotherapies, it is critical to develop effective non-opioid adjunctive therapies, which are widely available, present minimal side effects, and reduces the severity of OWS for individuals with OUD.

Abundant clinical evidence exists for the rapid and effective reduction in OWS through various neuromodulation approaches (Rosenthal 1972; Ellison et al. 1987; Qureshi et al. 2020; Young et al. 2020). In a randomized clinical trial, transcutaneous electrical acupoint stimulation (TEAS) was delivered as an adjunct to suboxone (Meade et al. 2010). TEAS was delivered at alternating low and high frequencies (2/100 Hz) for 30-minutes each day for three to four days. Two weeks post-discharge, the active TEAS group were 77% less likely to have used drugs, compared to 33% in sham treatment. In 2018, the FDA cleared a percutaneous electrical nerve field stimulator (PENFS) for OWS based on positive results from a retrospective study. Participants used the PENFS device during acute opioid detoxification to alleviate OWS without the use of prescription opioids (Miranda and Taca 2018). Although there was a clinically meaningful reduction in OWS, PENFS is limited in usability and patient compliance, which can significantly reduce therapeutic effectiveness. This device would later serve as predicate for FDA 510(k) clearance of another PENFS device (DyAnsys n.d.).

This clinical trial sought to investigate whether a novel and non-invasive transcutaneous auricular neurostimulation (tAN) device, developed to overcome the obstacles presented by PENFS (i.e., needle electrodes), can be used safely and effectively to reduce opioid withdrawal symptoms in support of an FDA clearance. It was hypothesized that activating auricular cranial nerve branches using tAN would confer a rapid and clinically meaningful reduction in opioid withdrawal symptoms, as defined by a 15% or greater reduction in COWS scores (Wesson and Ling 2003, Tompkins et al. 2009), without significant adverse effects.

## Methods

### Study design

**Fig. 1:**
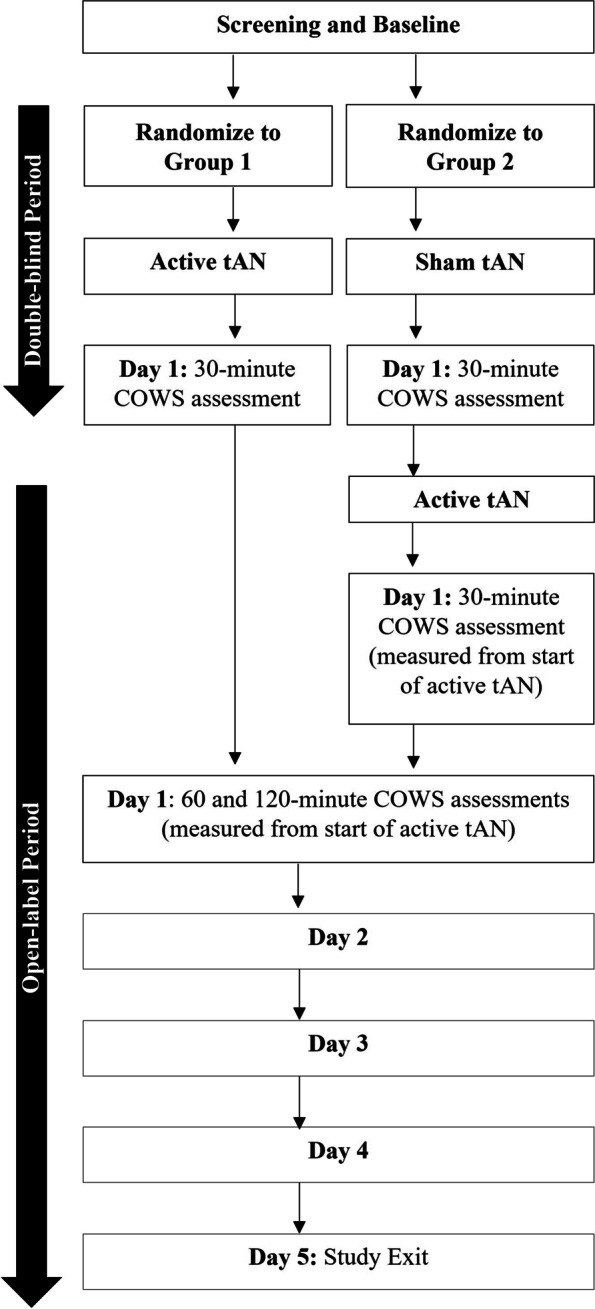
Clinical Study Design Diagram

The study design consists of a 30-minute randomized, sham-controlled, double-blind period followed by a 5-day open-label inpatient period as shown in Fig. 1. Participants were screened for trial eligibility after providing written informed consent and baseline measures were collected. Participants were then randomized 1:1 into either Group 1 or Group 2. Those assigned to Group 1 received active tAN in both the double-blind and open-label periods of the study. Subjects assigned to Group 2 received passive sham tAN (no stimulation) in the double-blind period, followed by active tAN in the open-label period. Participants in both groups were evaluated for opioid withdrawal symptoms, as measured by the COWS, at the completion of the 30-minute double-blind period. While the overall goal of the study was to assess a decrease in opioid withdrawal symptoms during acute detoxification, the initial double-blind period allowed for the assessment of the acute effects of active tAN compared to a sham tAN control. The duration of the double-blind period was limited to 30-minutes to minimize the pain and discomfort of abrupt opioid discontinuation in those receiving sham tAN in Group 2.

During the open-label period immediately following the double-blind period, participants in both groups received active tAN. Additional COWS assessments were collected at 60 and 120-minutes from the start of active tAN, which was at the beginning of the double-blind period for Group 1 and the end of the double-blind period for Group 2. All participants were followed for five days during inpatient treatment at a single site in the US.

### Participants

Individuals seeking OWS treatment were screened for study eligibility. All participants met the following eligibility criteria: current opioid physical dependence, use of prescription or non-prescription opioid; COWS score ≥ 13 at baseline or, in the opinion of the investigator, in moderate to severe withdrawal; 18–65 years of age; English language proficiency; able to provide informed consent and function at an intellectual level sufficient for study requirements. Participants with the following were excluded: current evidence of an uncontrolled and/or clinically significant medical condition; history of seizures or epilepsy; history of neurological diseases or traumatic brain injury; use of long-acting opioids such as methadone or buprenorphine for five or more consecutive days prior to enrollment; recent suicide attempt leading to current hospital admission or continued expressed suicidal ideation; presence of devices (i.e., pacemakers, cochlear prosthesis, neurostimulators); abnormal ear anatomy or ear infection present; need for concurrent treatment of alcohol or benzodiazepine withdrawal. Females who were pregnant or lactating or of childbearing potential, not using adequate contraception or not willing to comply with contraception for the duration of the study were also excluded.

### Randomization and blinding

Individuals were randomized using permuted block randomization with group size of four generated by the trial statistician (SW) following a baseline assessment. Sealed, opaque envelopes were provided to the clinical site and opened by the unblinded study coordinator. Active tAN or sham tAN devices were placed at the beginning of the double-blind period, for Groups 1 and 2 respectively. Participants in both groups were informed that “stimulation may or may not be perceived initially, since the Sparrow device will be preparing their neural system for long-term therapy.” Participants were blinded to group assignment until after the 30-minute COWS assessment was performed, which occurred at the completion of the double-blind period. The nursing staff providing care and performing the COWS assessment were blinded, which maintained a single-blind during the entire open-label period. The study statistician was unblinded to treatment during data analysis. No unblinding of COWS assessors occurred during the trial.

### Procedures

**Fig. 2:**
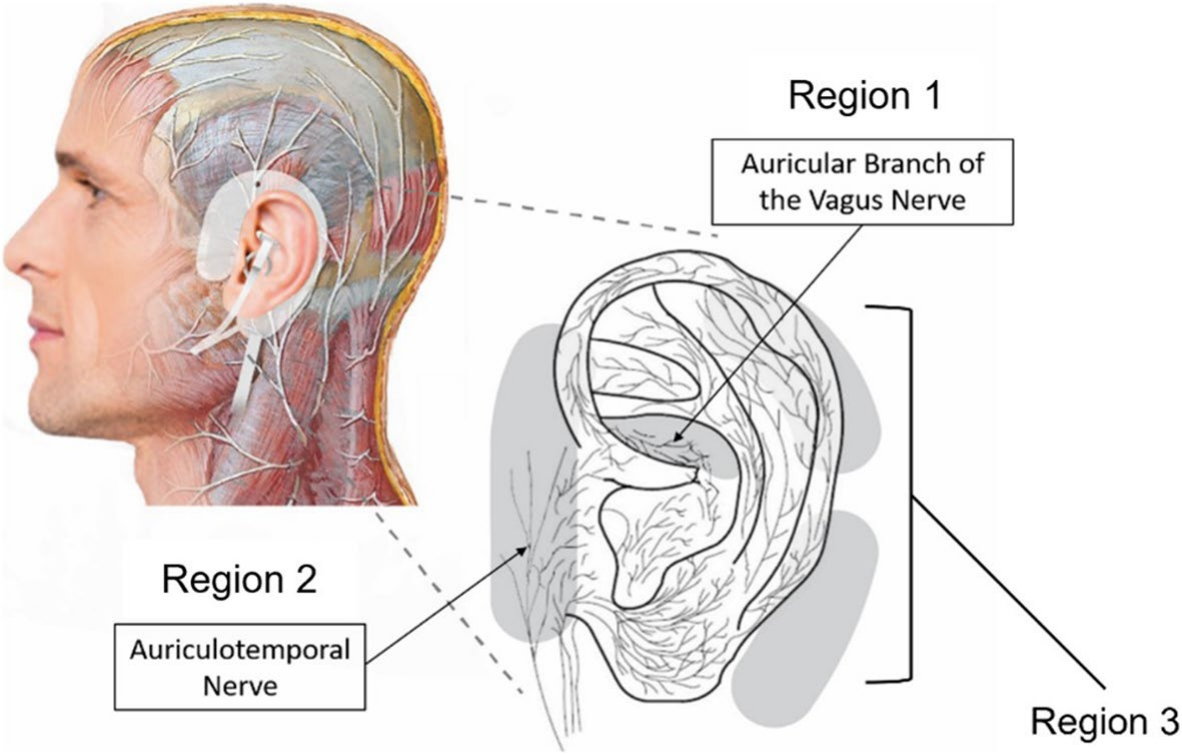
Form and Fit of tAN Device Around the Ear Neural Structures

Baseline measures were collected after participants were screened for trial eligibility. Blinded assessors performed a baseline COWS assessment, and participants completed three validated questionnaires: PHQ-9 to assess depression symptoms, PCL-5 to assess PTSD symptoms, and WHOQOL-BREF to assess overall quality of life. A urine drug screen was performed to confirm presence of opioids prior to treatment. Participants were then assigned to a treatment group and the Sparrow® device was applied (Spark Biomedical Inc., Dallas, TX). The device is a wearable, battery-operated system designed to provide transcutaneous stimulation on and/or around the auricle to treat OWS. The system can be worn up to 24-hours a day or as needed. A disposable earpiece containing four electrodes is applied to position the electrodes and stimulate three key dermatome regions (Fig. 2). These regions are adjacent to several cranial nerve branches. Specifically, the electrodes are located on the cymba concha (Fig. 2, one electrode - Region 1), the temporomandibular joint region, just anterior to the tragus (Fig. 2, one electrode - Region 2), and behind the auricle (Fig. 2, two electrodes - Region 3).

The dermatome areas corresponding to Regions 1 and 2 are targeted specifically based on the superficial subcutaneous nerves, while Region 3 encompasses the return electrodes. The subcutaneous tissue in Region 1 is innervated by the auricular branch of the vagus nerve (ABVN) (Peuker and Filler 2002). The ABVN arises from the superior vagal ganglion and is joined by the glossopharyngeal nerve (Standring 2015). The auriculotemporal nerve (ATN), which is a branch of trigeminal nerve, ascends subcutaneously from the location of electrode in Region 2. The ATN arises from the mandibular nerve and communicates with the facial nerve (Janis et al. 2010; Yang and Yoo 2014). The return electrodes in Region 3 are located superficial to the temporal and mastoid bone behind the auricle.

After earpiece placement, the device was programmed using a secured iOS device with a custom clinician programming application. Stimulation pulses for the two channels (Region 1 and 2) were interleaved; thus, even though both channels were ON, pulses from either channel did not overlap. The stimulation waveform was the same for all participants; square biphasic with a duration of 250 μs per phase and a 100 μs interval between phases. In order to minimize neural adaptation, stimulation was applied using a duty cycle of 5-minutes ON / 10 seconds OFF. The therapeutic stimulation intensity (mA) was increased to perceptive discomfort and lowered to comfort. Average stimulation intensity was 1.0 mA for Region 1 and 1.4 mA for Region 2.

The therapeutic paradigm included stimulation of both vagal and trigeminal nerve branches based on evidence that activation of these cranial nerves can be applied independently to treat the same conditions in more than one disease state: traumatic brain injury (Chiluwal et al. 2017; Neren et al. 2016), depression (Shiozawa et al. 2015; O'Reardon et al. 2006), epilepsy (Gil-López et al. 2020; Wheless et al. 2018), and migraine (Stanak et al. 2020; Evers 2021). This suggests that trigeminal and vagal stimulation share common effector pathways, which could lead to a potential synergetic effect (Cicco et al. 2018; Fanselow 2012). In application, low frequencies (≤ 25 Hz) have demonstrated efficacy for vagal stimulation, while higher frequencies (≥ 60 Hz) have demonstrated efficacy for trigeminal stimulation (Neren et al. 2016; O'Reardon et al. 2006; Gil-López et al. 2020; Wheless et al. 2018; Stanak et al. 2020; Evers 2021). Thus, the two individual stimulation frequencies were set: 5 Hz at cymba concha (Region 1/Channel 1; vagal innervation) and 100 Hz adjacently anterior to tragus (Region 2/Channel 2; trigeminal innervation).

As stated, participants in Group 1 began active tAN therapy at the start of the double-blind period and continued through the 5-day open-label period. Those in Group 2 were placed with the device at the start of the double-blind period but received sham tAN (no stimulation) until the end of the double-blind period. Group 2 then received active tAN for the 5-day open-label period using the same stimulation parameters as Group 1. After 120-minutes of active tAN, all participants were allowed to adjust their stimulation intensity based on participant tolerability (Region 1; mean 1.3 mA SD (0.8), Region 2; 2.2 mA (1.1). All participants received active tAN for up to 24-hours a day on Days 2–5.

In addition to baseline measurements, COWS scores were collected at 30, 60, and 120-minutes after start of active tAN for all participants on Day 1. Those assigned to Group 2 had an additional COWS assessment following 30-minutes of passive sham stimulation. Heart rate monitoring was performed using a Polar H10 heart rate sensor (Polar USA, Lake Success, NY) throughout study assessments on Day 1 and daily for up to 1-hour between 8:00 and 11:59 am on Days 2–5. COWS scores were captured daily during heart rate monitoring. Urine drug screening was repeated on Days 3 and 5 prior to participants undergoing a naloxone challenge. The challenge was not conducted if the participant tested positive for an opiate, to prevent precipitated withdrawal. tAN was discontinued approximately 1-hour prior to the naloxone challenge to prevent conflicting results. Participants passing the naloxone challenge on Day 3 could exit the study early. All participants returned the study device and exited the study on Day 5 after completing the PHQ-9, PCL-5, and WHOQOL-BREF. The study coordinator attempted to contact the participant seven days following study exit to assess the occurrence of any delayed device-related adverse events. Information related to all adverse events occurring after the participant provided informed consent were documented.

Use of opioid-based medications were not permitted at any point during the study. However, non-opioid-based medications (i.e., ancillary comfort medications) were permitted only after the participant completed the 60-minute COWS assessment after start of active tAN therapy. Commonly used comfort medications were ondansetron, methocarbamol, acetaminophen, diazepam, and clonidine. Comfort medications were not administered above normally prescribed levels, nor administer between 6:00 am and 11:59 am while COWS assessments were performed.

### Outcomes

The primary endpoint was change in COWS score from baseline to 60-minutes after start of active tAN therapy, pooled across all participants. A rapid relief of OWS improves the likelihood of therapy compliance. Secondary outcomes included change in COWS scores at 30-minutes (active tAN vs. sham tAN) to evaluate between-group differences in OWS reduction. Additionally, COWS scores following 120-minutes of active tAN, and Days 2–5. Exploratory outcomes included heart rate variability measured by R-R interval, and change to PHQ-9, PCL-5, and WHOQOL-BREF scores. Safety was assessed using the proportion of participants with a device-related adverse event.

### Statistical analysis

Sample size was determined based on the primary endpoint, mean percent reduction in COWS scores after 60-minutes of active tAN. A previous study of PENFS demonstrated a mean percent reduction in COWS of 84.6%, and an effect size of *d* = 3.4 (Peuker and Filler 2002). A more conservative approach for sample size calculation was implemented with an effect size of *d* = 1.0, corresponding to a 25% COWS score reduction. With 1:1 allocation and a statistical significance level of *p* < 0.05, 36 participants (18 per group) provided 90% power. Assuming an attrition rate of approximately 25%, we anticipated enrolling up to 45 participants and randomizing up to 40. The primary endpoint analysis was performed using the ITT population. An analysis was performed to determine whether data from both groups could be pooled for the primary efficacy analysis. All secondary and exploratory endpoints were analyzed only for participants with available data; no data was imputed. The safety population included participants who signed an informed consent form. Statistical analyses were performed using GraphPad Prism 9.1.0 (GraphPad Software, San Diego, CA).

A D’Agostino-Pearson test confirmed normality of the distribution of COWS scores for the primary endpoint, and a two-tailed paired t-test examined change in COWS scores from baseline to 60-minutes of active tAN. For the comparison of COWS score between Groups 1 and 2 after 30-minutes of active tAN versus sham tAN during the double-blind period, baseline factors were compared across groups to ensure no significant confounding factors were present, and scores were compared using a two-tailed, unpaired t-test with Welch’s correction. This analysis also determined if the groups could be pooled for the primary efficacy analysis. Change in COWS scores across time were analyzed using repeated measures analysis of variance in the ITT population and restricted maximum likelihood estimation for analysis without imputation with Dunnett’s post-hoc tests for pairwise comparisons. All exploratory endpoints were tested for normality using the D’Agostino-Pearson test and compared across time using paired t-tests or were analyzed using Chi-square or Fisher’s exact if categorical. This trial did not use a data monitoring committee.

## Results

**Fig. 3:**
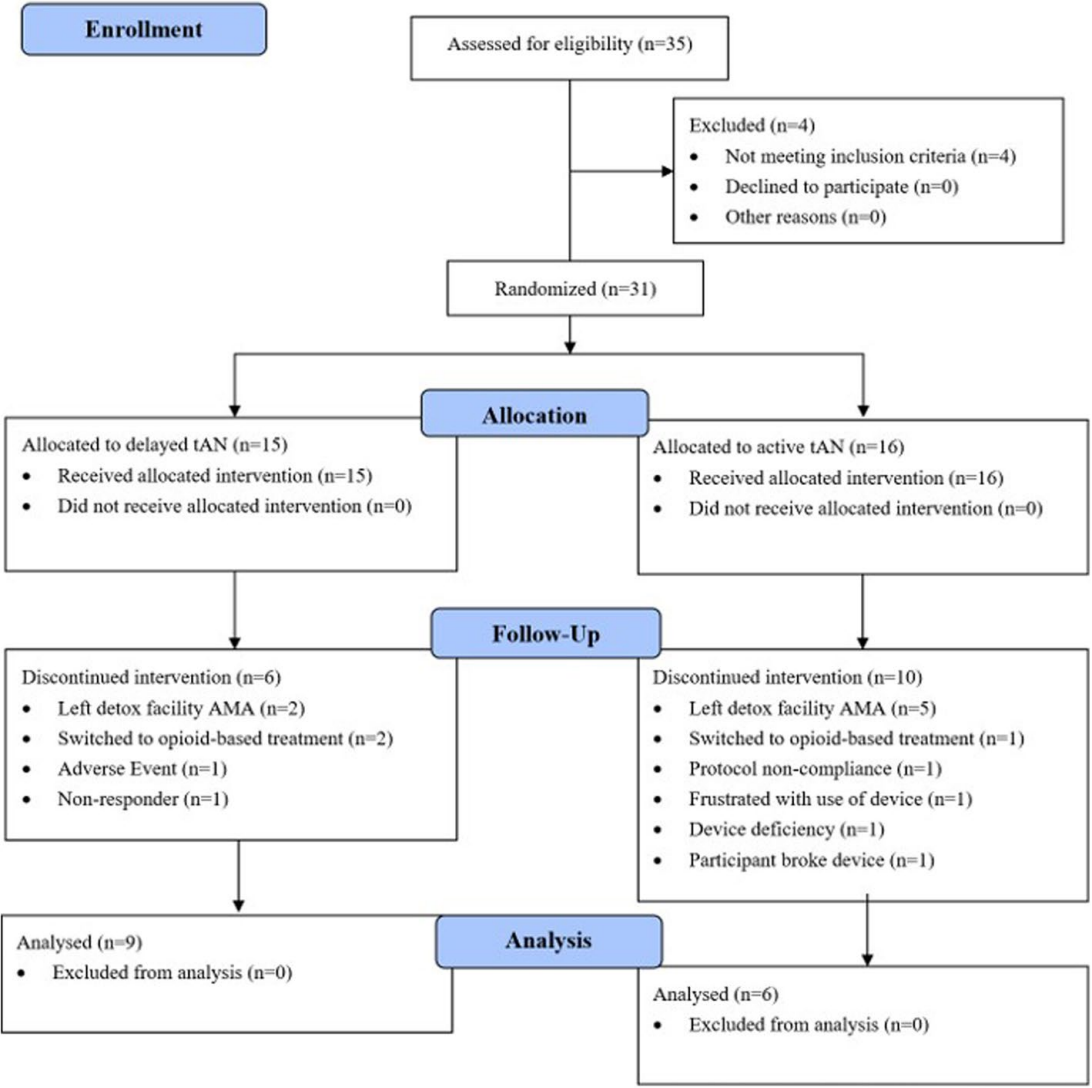
CONSORT Flow Diagram

**Fig. 4:**
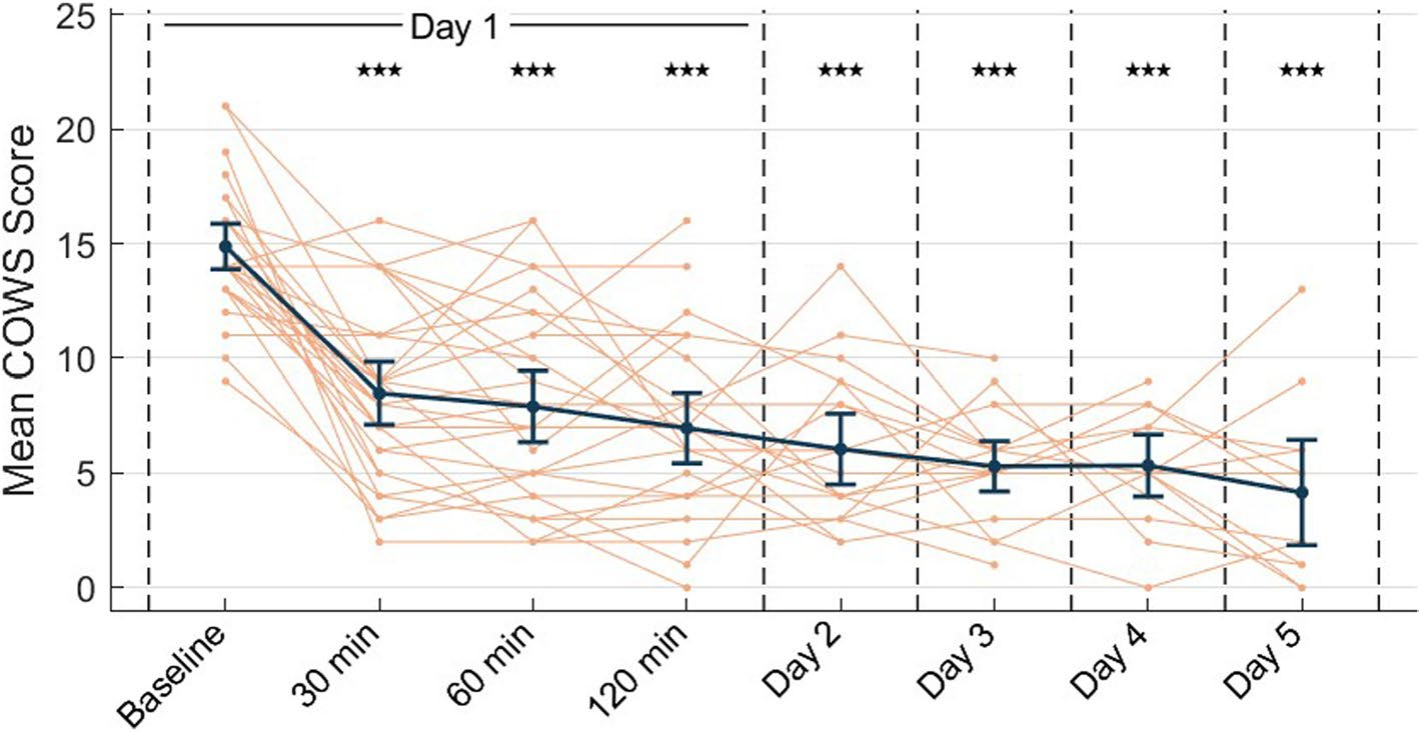
COWS Scores Across Time in the Pooled Population Without Data Imputation. Legend: *** denotes *p* < 0.0001. Timepoints denote the number of minutes from the start of active tAN, which is either at the start or end of the double-blind period, depending on group assignment. Dark blue line indicates the mean COWS score of study participants at each timepoint. Orange lines indicate individual participant COWS score at each timepoint

Thirty-five participants were enrolled between December 2019 and December 2020. Thirty-one participants were assessed for eligibility and randomly allocated equally to treatment (Fig. 3). Table 1 provides demographics and baseline characteristics. Across the two groups, the mean (SD) age at enrollment was 33.4 (7.3) years, 64.5% male, 83.9% Caucasian, and common co-morbidities included depression, anxiety, and bipolar disorder. The mean (SD) duration from last opioid use to initiation of tAN was 2.5 days (1.5). Table 2 and Fig. 4 illustrate reduction in COWS scores across time. Primary endpoint analysis showed the mean (SD) COWS score, pooled across both groups, was significantly reduced from baseline by 7.0 (4.7) points at 60-minutes after start of active tAN (95% CI, 4.2 to 9.7; < 0.0001; Cohen’s *d* = 2.0), demonstrating a significant and clinically meaningful percent reduction in COWS score of 45.9%. No significant differences were observed between groups for mean percent reduction in COWS score at 60 minutes after the start of active tAN (*p* = 0.668), thus determining groups could be pooled. Results were similar in the population for which data imputation was not conducted, suggesting that the pattern in the results was not driven by any particular data point.

**Table 1:** Baseline characteristics of the intention-to-treat population

	Group 1 (***n*** = 16)	Group 2 (***n*** = 15)	All (***N*** = 31)
**Mean**^**1 **^**(SD) Age at Enrollment (years) (Range)**	34.2 (7.3)	32.5 (7.4)	33.4 (7.3)
	(21–47)	(19–44)	
**Mean (SD) Baseline COWS score (Range)**	15.3 (2.7)	14.5 (2.7)	14.9 (2.7)
	(10–21)	(9–19)	(9–21)
**Mean (SD) Duration of Opioid Use (years) (Range)**	12.9 (7.0)	10.8 (6.4)	11.8 (6.7)
	(1–27)	(0.5–24.0)	
**Mean (SD) Morphine Milligram Equivalent (Range)**	1406.3 (1031.6)	1209.3 (566.5)	1311.0 (831.8)
	(300–4000)	(150–2000)	(150–4000)
**Gender: n (n/N%)**			
Female	6 (37.5%)	5 (33.3%)	11 (35.5%)
Male	10 (62.5%)	10 (66.7%)	20 (64.5%)
**Race: n (n/N%)**			
White	14 (87.5%)	12 (80.0%)	26 (83.9%)
Hispanic or Latino	1 (6.3%)	3 (20.0%)	4 (12.9%)
Black or African American	1 (6.3%)	0 (0%)	1 (3.2%)
**Psychiatric Co-Morbidities**^**2,3**^			
Depression	4 (25.0%)	6 (40.0%)	10 (33.3%)
Anxiety	3 (18.8%)	7 (46.7%)	10 (33.3%)
Bipolar Disorder	4 (25.0%)	4 (26.7%)	8 (26.7%)
**Opioid Type**^**2,4**^			
Heroin	14 (93.3%)	13 (86.7%)	27 (90.0%)
Prescription Narcotics	1 (6.7%)	4 (26.7%)	5 (16.7%)
Buprenorphine/Naloxone	1 (6.7%)	0 (0%)	1 (3.3%)
Methadone	0 (0%)	1 (6.7%)	1 (3.3%)
Fentanyl	0 (0%)	1 (6.7%)	1 (3.3%)
**Non-Opioid Drug Use in Previous 30 Days**^**2**^			
Alprazolam	5 (33.3%)	4 (26.7%)	9 (30.0%)
THC	3 (20.0%)	5 (33.3%)	8 (26.7%)
Alcohol	4 (26.7%)	5 (33.3%)	9 (30.0%)
Methamphetamine	5 (33.3%)	7 (46.7%)	12 (40.0%)
Cocaine	4 (26.7%)	2 (13.3%)	6 (20.0%)
Other	1 26.7%)	4 (26.7%)	5 (16.7%)

^1^Means analyzed using unpaired *t*-test with Welch’s correction and Chi Square or Fisher’s Exact test used for proportions

^2^Data not available for one participant in Group 1

^3^Participants may have more than one co-morbidity

^4^Participants may have used more than one opioid

**Table 2:** Change in COWS scores across time in intention-to-treat population and without data imputation

Timepoint^**1**^	Intention-to-Treat				Data Not Imputed					
	Mean (SD) Reduction	95% CI	Percent Change	p-value^**2**^	n	Mean (SD) Reduction	95% CI	Percent Change	p-value^**3**^	Percentage of Participants with Clinically Meaningful Reduction (n/N%)^**4**^
30 minutes	6.4 (4.2)	[3.9, 8.9]	42.0%	< 0.0001	31	6.4 (4.2)	[3.9, 8.9]	42.0%	< 0.0001	24 (77.4%)
60 minutes	7.0 (4.7)	[4.2, 9.7]	45.9%	< 0.0001	31	7.0 (4.7)	[4.2, 9.7]	45.9%	< 0.0001	26 (83.9%)
120 minutes	7.4 (4.3)	[4.9, 9.9]	49.6%	< 0.0001	27	7.6 (4.3)	[5.3, 11.0]	51.8%	< 0.0001	25 (92.6%)
Day 2	7.5 (4.4)	[4.9, 10.0]	50.0%	< 0.0001	20	8.1 (4.0)	[6.0, 12.0]	56.3%	< 0.0001	19 (95.0%)
Day 3	7.9 (4.1)	[5.5, 10.0]	53.3%	< 0.0001	20	8.8 (3.4)	[7.2, 12.0]	61.5%	< 0.0001	20 (100%)
Day 4	8.1 (4.2)	[5.6, 11.0]	54.5%	< 0.0001	15	9.9 (3.3)	[7.3, 12.0]	66.2%	< 0.0001	16 (100%)
Day 5	8.4 (5.0)	[5.5, 11.0]	56.1%	< 0.0001	13	10.7 (4.9)	[6.5, 15.0]	70.4%	< 0.0001	12 (92.3%)

^1^Timepoints denote number of minutes after start of active tAN

^2^Repeated measures ANOVA

^3^Mixed-effects model (REML)

^4^Clinically meaningful reduction defined as a 15% or greater reduction in score

**Fig. 5:**
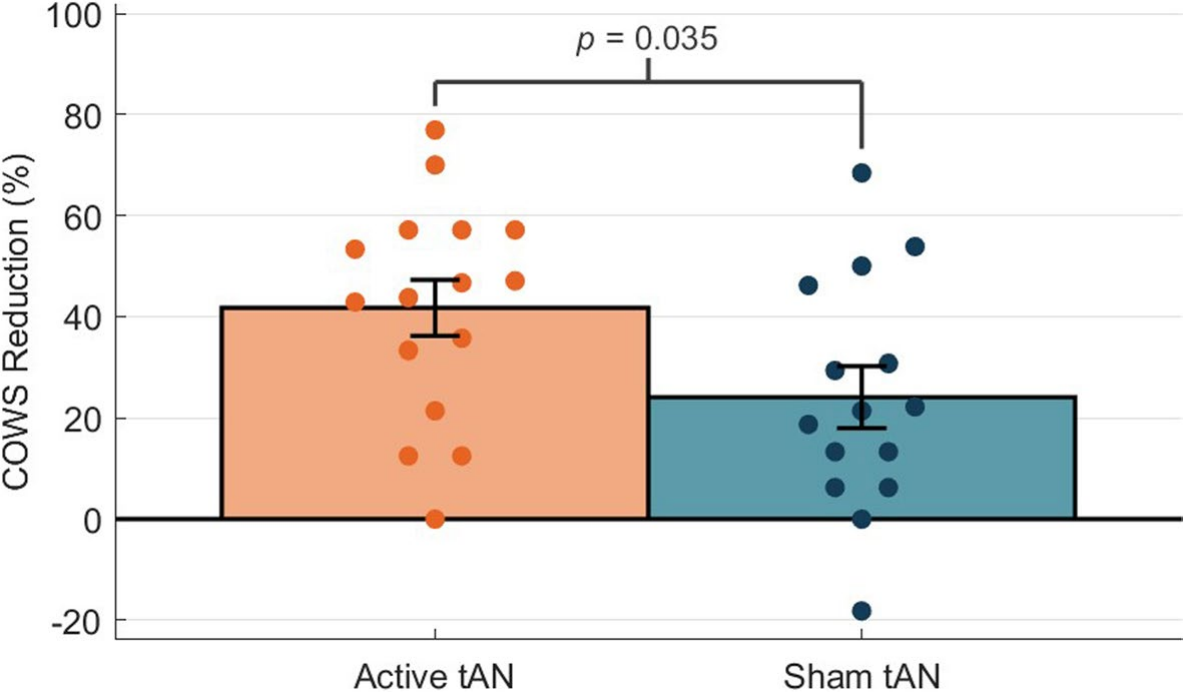
Comparison of COWS Scores: Active and Sham tAN at the end of the double-blind period. Legend: *P*-value; Two-sided independent Welch’s t-test; Data representing the intent-to-treat population. Group 1 received active tAN and Group 2 received sham tAN (no stimulation) during this period. Colored dots indicate individual COWS score percent reductions for the respective treatment groups

Furthermore, the mean (SD) reduction in COWS score at the end of the 30-minute double-blind period was 3.7 (3.8) in the sham tAN group and 6.3 (3.2) in the active tAN group. This difference in mean percent reduction was significantly different (24.1% vs 41.7%, t(29) = 2.201, *p* = 0.036, Fig. 5). During the open-label period, COWS scores were further reduced by 7.4 (4.3) points after 120-minutes of active tAN, by 7.5 (4.4) points on Day 2, by 7.9 (4.1) points on Day 3, 8.1 (4.2) points on Day 4, and 8.4 (5.0) points on Day 5, corresponding to a 49.6, 50.0, 53.3, 54.5, and 56.1% reduction. The overall RMANOVA yielded significance in COWS scores across time in the ITT population (F(7, 210) = 36.0, *p* < 0.0001), and pairwise comparisons yielded significant reductions at each time point when compared to baseline (Table 2).

Mean (SD) heart rate was significantly reduced from 83.2 (11.2) bpm at baseline to 78.5 (12.7) bpm 30-minutes following initiation of active tAN (*p* = 0.01) and mean (SD) R-R interval was significantly increased to 787.7 (128.6) milliseconds at the same timepoint (*p* = 0.003), suggesting tAN may have acute autonomic effects (increased parasympathetic activity) in this patient population. No significance was recorded at subsequent timepoints, indicating a transient effect.

PHQ-9 and PCL-5 scores were significantly reduced from baseline to Day 5 (*p* = 0.0074 and *p* = 0.0013; Table 3). PHQ-9 scores were reduced by 5.1 (6.0) points and PCL-5 scores were reduced by 9.8 (8.9), corresponding to a 35.0% reduction on the PHQ-9 and 32.7% reduction on the PCL-5. No significant change was observed in the WHOQOL-BREF domain scores (Table 4). Most participants who qualified (negative for opiates on urine drug screen) and completed the naloxone challenge (11/12; 91.7%) passed, indicating these participants could start opioid antagonist medication (i.e., Vivitrol) on Day 5.

**Table 3:** Change in PHQ-9 and PCL-5 scores at Day 5 in the population without data imputation

Questionnaire	n^**1**^	Mean (SD) Reduction	95% CI	Percent Reduction	P value^**2**^	Percentage of Participants with Clinically Meaningful Reduction (n/N%)^**3**^
PHQ-9	14	5.1 (6.0)	[8.532, 1.611]	29.5 (35.0)%	0.0074	6 (42.9%)
PCL-5	14	9.8 (8.9)	[14.95, 4.621]	29.8 (32.7)%	0.0013	5 (35.7%)

^1^Excludes one participant with score over 2 standard deviations above the mean

^2^Two-tailed, paired t-test

^3^Defined as at least a 5-point decrease for PHQ-9 and at least a 10-point decrease for PCL-5

**Table 4:** Change in (transformed 0–100) WHO-QoL-BREF scores at Day 5 in the population without data imputation

Domain	n^**1**^	Mean (SD) Increase	95% CI	P value^**2**^
Physical Health	14	10.5	[−6.325, 27.24]	0.2013
Psychological Health	14	6.5	[−11.40, 24.49]	0.4446
Social Relationships	14	17.3	[−3.281, 37.80]	0.0926
Environment	14	12.3	[−4.964, 29.52]	0.1479

^1^Excludes one participant with score over 2 standard deviations above the mean

^2^Two-tailed, paired t-test

Additional post-hoc analyses of COWS score demonstrated that 26/31 (83.9%) participants were categorized as in mild withdrawal (COWS < 13), and 8/31 (25.8%) participants demonstrated no withdrawal (COWS < 5) at 60-minutes after the start of active tAN.

Two participants (7.7%) experienced skin irritation near the cymba concha. Both device-related adverse events were mild and required no medical intervention for resolution.

## Discussion

OWS is commonly described as unbearable, and avoidance of withdrawal is one of the principal factors driving resumption of opioid use. Individuals seeking to abstain from opioids often fail to complete opioid withdrawal treatment due to the overwhelming nature of OWS (Srivastava et al. 2020). Conventional agonist and partial agonist pharmacotherapies to mitigate OWS are effective, but also carry the risk of severe OWS upon discontinuation leaving few effective options for individuals who do not continue an opioid indefinitely for OUD (Stotts et al. 2009). Here, are the results of a clinical investigation of a non-invasive and non-opioid treatment option to treat OWS. To the best of our knowledge, this is the first prospective clinical trial to demonstrate a rapid and clinically meaningful effect with a 45.9% reduction in OWS after 60-minutes of active tAN. In addition, participants achieved an average reduction up to 61.0% on Days 2–5 in the ITT population, and 74.7% in not imputed data.

These results exceed those from clinical trials of other approved and off-label medications for acute opioid withdrawal, specifically after one day of treatment. Among these pharmacotherapies, the mean percent reduction in COWS was less than 30% in a 24-hour timespan (Dunn et al. 2017; Fishman et al. 2019) and common side effects included hypotension and bradycardia. Regarding non-pharmacological therapies, PENFS provided results from a retrospective analysis in which the device was used off-label on patients with moderate to severe OWS (Miranda and Taca 2018). Although clinically meaningful reductions in symptoms were observed with PENFS after 60-minutes, the percutaneous device is limited in usability and compliance. PENFS requires placement of needle electrodes through transillumination by a trained health care provider and any incidental electrode displacement renders the device ineffective, requiring additional provider intervention. Non-invasive tAN therapy resulted in similar or better management of acute OWS in the absence of any cardiovascular side effects and with improved usability when compared to PENFS. Furthermore, tAN therapy demonstrated a monotonically decreasing effect for the entire duration of the therapy without any withdrawal symptoms rebound.

During the 30-minute randomized, sham-controlled, double-blind period, active tAN demonstrated a significantly greater reduction in COWS scores compared to sham tAN. Although the double-blind period of the study occurred over a short time window to test acute effectiveness and potential placebo effect, the results were clinically meaningful. Demonstrating no placebo effect during early stages of opioid withdrawal is critical, as peak withdrawal symptoms typically present on Days 2–3, thus reducing the likelihood of any sustained placebo effect during the treatment time course.

Furthermore, tAN demonstrated significant improvements related to symptoms of depression and PTSD. Nearly half of patients experienced a clinically meaningfully reduction in depression symptoms, and approximately one-third of patients experienced a clinically meaningful reduction in PTSD symptoms. This evidence suggests that tAN may target, in many cases, the underlying cause of addiction: depression, PTSD, trauma, and overall mental dysfunction (Koob 2020). Additionally, in a prospective, open-label trial, tAN demonstrated to be a safe and effective adjuvant treatment to oral morphine therapy in infants suffering from neonatal opioid withdrawal syndrome (Jenkins et al. 2021).

tAN demonstrated acute significant reductions in heart rate and significant increases in R-R interval. These data suggest that tAN modulates the vagal parasympathetic nervous system. OWS typically manifest as high sympathetic activity, thus promoting parasympathetic activity may lead to restoration of autonomic balance.

The proposed mechanism for tAN is based on pre-clinical and clinical research suggesting that the therapeutic effects of vagus and trigeminal nerve stimulation are related to increased parasympathetic activation and release of endogenous opioids (endorphins) (Jenkins et al. 2021). tAN therapy targets stimulation of the left AVBN and ATN. Functional magnetic resonance imaging studies demonstrate that ABVN stimulation promotes activation of the nucleus tractus solitarius, locus coeruleus, spinal trigeminal nucleus, parabrachial area, periaqueductal gray, amygdala, and nucleus accumbens (Qureshi et al. 2020). Direct electrical stimulation to the parabrachial area and arcuate horn regions may trigger the release of endogenous opioids, resulting in analgesic effects, where the type of endogenous opioid released is dependent on stimulation frequency (Han and Wang 1992). As stated in Procedures, both vagal and trigeminal nerve branch stimulation have demonstrated therapeutic benefit for pain and other therapies (Mercante et al. 2018). Interestingly, both vagal and trigeminal afferents synapse on to the periaqueductal gray (Mercante et al. 2018), stimulation of which, in humans, demonstrate release of endogenous opioids (Sims-Williams et al. 2017). Given these findings, we hypothesized that vagal and trigeminal activation would synergistically mediate endogenous opioid release. However, this clinical trial was not designed to test these two working hypotheses. Further investigation into physiological biomarkers of OWS and the mobilization of endogenous opioids may optimize tAN therapy.

This study is not without limitations that should be addressed. A sample size of 31 patients is generally regarded as relatively small, as compared to pharmaceutical studies. A formal sample size calculation was performed based on results from the previous PENFS study (Miranda and Taca 2018). The effect size for COWS score reduction following PENFS is considered large (d = 3.4). A sample size calculation based on this effect size would have required a total of four participants in the current study. However, a more conservative approach was taken in our calculation and powered for a smaller effect size (d = 1.0), which corresponded to approximately a 25% COWS score reduction. With 1:1 allocation and a statistical significance level of *p* < 0.05, 36 participants (18 per group) provided 90% power for detecting a benefit of treatment. Assuming an attrition rate of approximately 25%, we anticipated enrolling up to 45 participants and randomizing up to 40. An interim analysis which included a sample size re-estimation after 31 participants completed the study, determined this number of patients was sufficient for detecting a treatment benefit, and enrollment was halted to conserve resources.

An additional study limitation is the duration of the 30-minute double-blind period in which Group 1 received active tAN treatment and Group 2 received sham tAN treatment. Future trials that aim to determine the effectiveness of tAN therapy would further benefit with a control group that extends the entire duration of the treatment phase.

Four participants, during the open-label phase of the trial, were given non-opioid-based medication assisted therapies (MATs) (i.e., comfort medications) before they completed the 60-minute COWS assessment. An analysis of change in COWS score for these four participants confirmed these medications did not impact the assessments. Mean reduction in these participants was lower than mean reduction in those participants who did not receive any medication (6.0-point reduction compared to 8.8-point reduction). Despite these limitations, the study design and use of the ITT principle in analyses helped mitigate the risk of bias. The results from this trial were used to support FDA clearance of tAN. Thus, the design should be viewed as an adequate option for this patient population and the results indicate the therapy produces a meaningful therapeutic effect.

## Conclusions

This is the first study demonstrating the effects of tAN for patients experiencing OWS following abrupt opioid discontinuation. Across all study participants, tAN demonstrated to be safe, well-tolerated, and delivered clinically meaningful, rapid, and sustained reductions in opioid withdrawal symptoms. tAN therapy is one of three FDA cleared/approved non-opioid treatment options for OWS (tAN and Lofexidine) and OUD (extended-release injectable naltrexone) management. Thus, tAN therapy provides patients and healthcare professionals a novel, safe, and effective tool to aid in OWS treatment.

## Data Availability

The datasets used and/or analyzed during the current study are available from the corresponding author on reasonable request.

## Abbreviations

ABVN: Auricular Branch of the Vagus Nerve
ATN: Auriculotemporal Nerve
bpm: Beats per Minute
COWS: Clinical Opiate Withdrawal Scale
FDA: Food and Drug Administration
ITT: Intent to Treat
mA: Milliamp
MAT: Medication Assisted Therapy
OUD: Opioid Use Disorder
OWS: Opioid Withdrawal Syndrome
PCL-5: PTSD Checklist for DSM-5
PENFS: Percutaneous Electrical Nerve Field Stimulator
PHQ-9: Personal Health Questionnaire
PTSD: Post-Traumatic Stress Disorder
SD: Standard Deviation
tAN: Transcutaneous Auricular Neurostimulation
TEAS: Transcutaneous Electrical Acupoint Stimulation
WHOQOL-BREF: World Health Organization Quality of Life (Brief)
